# Vulvar Malignancy in Neurofibromatosis Syndrome

**DOI:** 10.1155/2013/217924

**Published:** 2013-09-19

**Authors:** Angela Musella, Innocenza Palaia, Lavinia Domenici, Assunta Casorelli, Angela Martoccia, Pierluigi Benedetti Panici

**Affiliations:** Department of Obstetrics and Gynecology, “Sapienza” University, Viale del Policlinico 155, 00155 Rome, Italy

## Abstract

Type 1 neurofibromatosis (NF1) is a dominantly inherited neurologic disorder that affects primarily the skin, bones, and peripheral nervous system. It may be associated with a variety of clinical manifestations including cafe-au-lait spots, skinfold freckling, Lisch nodules, and visceral neurofibromas. Individuals affected by NF1 harbor an increased risk for both benign and malignant tumors. Malignant transformation is usually observed in the form of neurosarcoma. Rarely, NF1 affects the genital tract, and isolated vulvar localization is extremely rare. Here is reported a rare case of a solitary neurosarcoma of the vulva in a 43-year-old woman affected by NF1 syndrome treated with surgical excision. The purpose of this case is to underline the possibility of association between NF1 and genital tract sarcoma and to suggest an accurate evaluation of rapid growth vulvar mass in this setting.

## 1. Introduction

Type 1 neurofibromatosis is the most common autosomal dominant phakomatoses, with an incidence of 1 in 2500–3000 births. Most of cases are hereditary but about 30% arise de novo from spontaneous genetic mutations [1].

Neurofibromatosis consists of at least two distinct dominantly inherited disorders: type 1 (NF1) that is responsible for about 85–90% of cases and type 2 (NF2) that was recently recognized as separate entities with responsible genes localized in chromosomes 17 and 22, respectively [2, 3]. 

Classic features of the clinical phenotype include the presence of café-au-lait spots, neurofibromas, axillary and inguinal freckling, Lisch nodules (hamartomas), and deformities of the skeletal system, as well as the risk of developing multiple tumors, especially in the central nervous system [4]. Neurofibromatosis rarely involves the genital tract including the vulva [5].

Individuals affected by NF1 harbor an increased risk for both benign and malignant tumors. Malignant transformation is usually observed in the form of neurosarcoma [6, 7]. Tumors in peripheral nerves are neurofibrosarcomas, malignant schwannomas, or malignant peripheral nerve sheath tumors. The literature data report an incidence of 7 to 13% of these neoplastic lesions in patients with neurofibromatosis [8]. Nevertheless in the higher risk for neuronal sarcomas, few cases of nonneurologic sarcomas in association with neurofibromatosis have been reported in the literature [9, 10]. To our knowledge, no cases of vulvar sarcoma in patients affected by von Recklinghausen syndrome have been described until now.

We report the case of a solitary vulvar sarcoma in a woman with NF1.

## 2. Case Presentation

A 43-year-old Caucasian woman referred to our department because of an enlarging, bleeding, and painful vulvar mass. She has a diagnosis of NF1 since the age of 22 based on findings of multiple café-au-lait spots and subcutaneous nodules. Patient reported a mild asymmetry of the vulva since two years but she has noted a rapid enlargement of the lesion during the last month.

On gynecological examination, the mass was localized on the right major labium of the vulva, measuring 15 centimeters in diameter, presenting a smooth surface and hard consistency, and was painful and easily bleeding. Urethral meatus, clitoris, vagina, cervix, and uterus appeared not to be involved. No enlarged inguinal lymph nodes were detected at palpation.

The patient underwent preoperative evaluation including chest X-ray, ECG, routinary blood exams, anesthesiologic evaluation, and a magnetic resonance (MR) that confirmed the characteristics of the vulvar lesion. MR imaging revealed a 12 × 10 × 10 centimeter in size, parenchymatous mass without evidence of stromal invasion. Hepatic lesions highly suspected for metastasis were detected. No other pathological cerebral lesions were found at cerebral MR. However, a surgical resection of the lesion to reduce patient discomfort was needed. 

**Figure 1:**
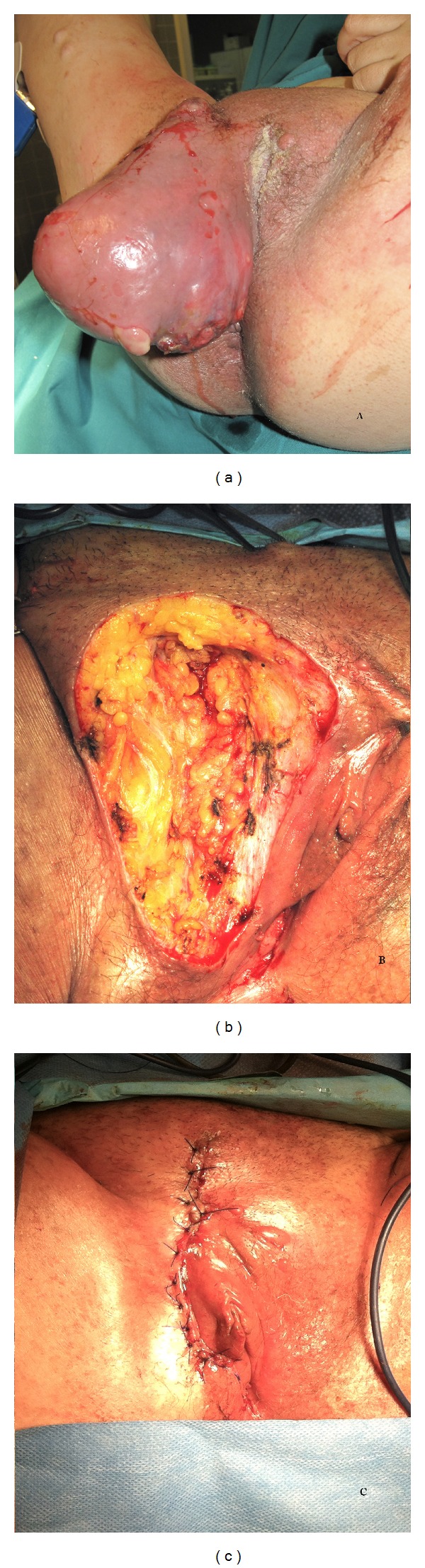
Vulvar sarcoma.

After informed consent, she underwent right hemivulvectomy with total resection of the mass (Figure 1). 

No intraoperative consultation or frozen section was performed. Histological evaluation revealed a vulvar carcinosarcoma with positivity for vimentin and focally positive for cytokeratin at immunohistochemistry analysis. Surgical margins were clear and distant about 30 millimeters from the neoplasia. No perioperative complications were reported. 

Adjuvant chemotherapy including 6 cycles of doxorubicin 60 mg/mq every 21 days and ifosfamide 10 g/mq for 4 days associated with mesna (AIM) every 21 days started 2 months after surgery. There is no evidence of recurrent disease on MRI of the chest, abdomen, and pelvis or on physical examination 3 months after surgery. Cosmetic and functional outcome at 4 months followup was good.

## 3. Discussion

Neurofibromatosis is a hereditary neurologic disorder firstly described by the German pathologist Frederich von Recklinghausen in 1882 [11]. 

NF1 is caused by neurofibromin-1 gene mutations. Neurofibromin-1 is involved in the control of cellular proliferation by stimulating the intrinsic GTP-ase of p21-ras. Loss of function in NF1 results in reduced control of cell proliferation [12]. For this reason, patients with NF1 have four-time greater probability of developing benign and malignant tumors [13]. Most frequently patients may develop a variety of central nervous system abnormalities. Neurofibromas can develop in all body, but female genital tract involvement is unusual. Genital NF1 presents such as a asymmetric, distorted external genitals. Vulva seems to be most frequently affected, with rare reports of vaginal, cervical, uterine, and ovarian involvement in patients with known von Recklinghausen disease [14].

Case reports and case series have documented female genital neurofibromas, most commonly affecting clitoris with other areas of the genitourinary system or rarely the labium majors alone. 

Clinical presentation of genital neurofibromas is generally associated with pain, bleeding, and functional impairment, especially for advanced and infiltrating lesions [15].

Diagnostic imaging is very important to evaluate the distinction between superficial and invasive tumors, and MR is the technique of choice to planning surgical resection of the lesion [16, 17].

Management of this kind of lesions includes a multidisciplinary approach involving the gynecologist, plastic surgeon, dermatologist, and histopathologist. If the tumor is suitable of surgery, a surgical excision with wide margins of 3 to 5 cm of normal skin should be performed [18].

Our patient presented a fast-growing cutaneous lesion, so the diagnosis was relatively early. 

Clinical management in this kind of patients was influenced by multiple factors such as age of patient, comorbidity, histopathological findings, size, location, and margins of the lesion. 

The comparison with other similar cases reported in the literature is difficult due to the paucity of series and the wide heterogeneity of cases relative to the original definition of histology and stage at presentation. Except for some cases diagnosed at early stage disease [19, 20], the vast majority of cases experienced a rapidly progressive and fatal disease [21, 22] despite the employment of multimodal treatment with radical surgery and adjuvant radiotherapy and/or chemotherapy.

Female genital tract sarcomas are very rare; moreover, no cases of vulvar sarcoma in patients with NF1 have been ever described. For this reason, the purpose of our case is to underline the possibility of this association and to suggest an accurate evaluation of rapid growth vulvar mass in this setting.
